# Direct cost associated with acquired brain injury in Ontario

**DOI:** 10.1186/1471-2377-12-76

**Published:** 2012-08-17

**Authors:** Amy Chen, Ksenia Bushmeneva, Brandon Zagorski, Angela Colantonio, Daria Parsons, Walter P Wodchis

**Affiliations:** 1 Toronto Rehabilitation Institute, Toronto, ON, Canada; 2 Institute of Health Policy Management and Evaluation, University of Toronto, Toronto, ON, Canada; 3 Institute for Clinical Evaluative Sciences, Toronto, ON, Canada; 4 Department of Occupational Science and Occupational Therapy, University of Toronto, Toronto, ON, Canada

## Abstract

**Background:**

Acquired Brain Injury (ABI) from traumatic and non traumatic causes is a leading cause of disability worldwide yet there is limited research summarizing the health system economic burden associated with ABI. The objective of this study was to determine the direct cost of publicly funded health care services from the initial hospitalization to three years post-injury for individuals with traumatic (TBI) and non-traumatic brain injury (nTBI) in Ontario Canada.

**Methods:**

A population-based cohort of patients discharged from acute hospital with an ABI code in any diagnosis position in 2004 through 2007 in Ontario was identified from administrative data. Publicly funded health care utilization was obtained from several Ontario administrative healthcare databases. Patients were stratified according to traumatic and non-traumatic causes of brain injury and whether or not they were discharged to an inpatient rehabilitation center. Health system costs were calculated across a continuum of institutional and community settings for up to three years after initial discharge. The continuum of settings included acute care emergency departments inpatient rehabilitation (IR) complex continuing care home care services and physician visits. All costs were calculated retrospectively assuming the government payer’s perspective.

**Results:**

Direct medical costs in an ABI population are substantial with mean cost in the first year post-injury per TBI and nTBI patient being $32132 and $38018 respectively. Among both TBI and nTBI patients those discharged to IR had significantly higher treatment costs than those not discharged to IR across all institutional and community settings. This tendency remained during the entire three-year follow-up period. Annual medical costs of patients hospitalized with a brain injury in Ontario in the first follow-up year were approximately $120.7 million for TBI and $368.7 million for nTBI. Acute care cost accounted for 46-65% of the total treatment cost in the first year overwhelming all other cost components.

**Conclusions:**

The main finding of this study is that direct medical costs in ABI population are substantial and vary considerably by the injury cause. Although most expenses occur in the first follow-up year ABI patients continue to use variety of medical services in the second and third year with emphasis shifting over time from acute care and inpatient rehabilitation towards homecare physician services and long-term institutional care. More research is needed to capture economic costs for ABI patients not admitted to acute care.

## Background

Acquired Brain Injury (ABI) from both traumatic (such as falls and motor vehicle crashes) and non-traumatic (such as anoxia and brain tumors) causes is a leading cause of death and disability in North America [1,2]. An acquired brain injury is damage to the brain after birth and is not due to a congenital disorder developmental disability or a process which progressively damages the brain [3]. The National Center for Injury Prevention and Control estimated that 2% of US population live with disability as a result of brain injury from traumatic causes alone [4]. Annually 1.7 million people in the US sustain Traumatic Brain Injury (TBI) resulting in 275000 hospitalizations and 52000 deaths [5].

ABI patients have long-lasting impairments and their treatment requires significant health care resources [6-9]. Evidence suggests that utilization of medical and rehabilitation services by TBI patients remains high for several years post-discharge [10-13]. Cameron and colleagues [13] matched patients with TBI with a non-injured comparison group and found that the TBI cohort had more post-injury hospitalizations (rate ratio (RR) = 1.54 95% CI = 1.39-1.71) greater cumulative lengths of stay (RR = 5.14 95% CI = 3.29-8.02) and a greater post-injury physician claims rate (RR = 1.44 95% CI = 1.35-1.53) than the non-injured cohort. Advances in medical science in recent years may have resulted in better outcomes and higher survival probability for ABI patients with more patients directed to rehabilitation centers to augment their recovery [14]. However TBI patients admitted to the post-acute settings are medically complex have longer LOS and are at increased risk of re-hospitalization. The Canadian Institute of Health Information (CIHI) reported that the median length of stay (LOS) in rehabilitation centers for patients with brain dysfunction 36 days versus 21 days for average rehabilitation patient [15]. Patients with head injuries also had prolonged stays in other sub-acute facilities with the median LOS in complex continuing care of 92 days versus 40 days for all patients cared for in the same setting [16].

Despite the substantial financial burden of ABI there is limited research on the comprehensive costs associated with it. Significant attention has been devoted to estimating costs of TBI but this literature is subject to a number of limitations. In particular many studies examine only a subset of TBI patients such as patients with a specific level of injury severity [17,18] occupation type [19] or a specific age [20]. Other findings are based on a small non-representative sample of patients [10 11 20] and the authors use the incidence rates to project sample-based cost estimates to the entire population. Some authors relied on patients’ recall to identify their use of health services [10] or used systematic reviews of TBI costing literature [21,22]. In addition most studies only covered costs related to the initial hospitalization [17,21-24]. Given that hospitalized TBI patients often face a prolonged recovery process and suffer from long-lasting consequences of injury [9] such an approach clearly underestimates the true burden of TBI-related costs. Only a handful of studies reported costs across a continuum of care [19,20] none of which were based on recent Canadian data. Past studies have focused exclusively on TBI and comparator heath system costs of non-traumatic brain injury (nTBI) have not been previously estimated.

The current study aims to address limitations of previous research. The present findings are based on recent and comprehensive data. The study examines health system burden in terms of direct costs to the public health care system for all ABI patients discharged from Ontario acute care hospitals between the years 2004–2008. Since Ontario is the most populous Canadian province representing 38% of Canadian population patient records analyzed in this study comprise a large and representative sample of the Canadian population of ABI patients which makes our results generalizable to the Canadian population. The findings can also be extended to other jurisdictions assuming that they share epidemiology and treatment patterns that are similar to the ones in Ontario. Ontario administrative data have been shown to provide reliable records of utilization of a variety of health care services based on data from provincial administrative healthcare databases [25-27]. In addition to estimating the medical costs of TBI we expand the previous studies by estimating direct costs for nTBI patients. The economic costs for TBI and nTBI are further broken down into those discharged to rehabilitation and those who were not. Rehabilitation is a costly service that is most often used for those who are most severely injured and suffer from more severe functional impairment. Therefore the results are presented this way to show costs for those with greater functional impairments. The primary objective of this study is to provide accurate and comprehensive estimates of direct medical costs from initial hospitalization up to 3 years post-injury among individuals with ABI for the years 2004–2008.

## Methods

### Study population

The study population consisted of all patients discharged alive from acute care with either traumatic or non-traumatic brain injury between April 1 2004 and March 31 2007. Patients in the cohort were followed retrospectively until the earlier of death or March 31 2008 and were stratified according to the cause of brain injury (TBI and nTBI) and their discharge disposition from an acute care hospital (inpatient rehabilitation or elsewhere). We identified 11970 cases of TBI and 31501 cases of nTBI using patients’ associated ICD-10 codes in any diagnosis position in the Discharge Abstract Database (DAD) which records all hospital admissions in the province of Ontario and up to 25 diagnoses. Patients with the following diagnoses were classified into TBI category: fracture of skull intracranial injury late effects of injuries poisonings and toxic effects and other external causes. Patients with a stroke in any diagnosis position and patients with hospital-acquired ABI diagnosis were excluded. Diagnoses in nTBI group corresponded to the following ICD-10 codes: toxic effects of substances complications of surgical and medical care not elsewhere classified anoxia vascular insults brain tumours encephalitis metabolic encephalopathies and meningitis. The nTBI group did not include patients with a stroke as their most responsible diagnosis since this population represents a well-studied and unique impairment. The exact ICD-10 codes are reported in Table 1. To ensure that we have identified new cases rather than readmissions patients who suffered from ABI in the year prior to their index injury were excluded from the analysis.

**Table 1:** ICD Codes for Conditions leading to Acquired Brain Injury (ABI)Final List of Codes based on Feedback and Studies –Ontario Neurotrauma Foundation ABI Pilot Project

**I. Traumatic Causes**	
**Diagnosis**	**ICD-10 Code and Description**
1. Fracture of the skull	S02.0 Skull
	S02.1 Base of skull
	S02.9 Skull and facial bones S02.7 Multiple fractures involving skull and facial bones
	S02.89 Fractures of other unspecified skull and facial bones
2. Intracranial Injury excluding those with skull fracture	S06.0 Concussion
	S06.2 Diffuse brain injury
	S06.4 – Epidural Hemorrhage
	S06.5 – Traumatic subdural hemorrhage
	S06.6 – Traumatic subarachnoid hemorrhage
	S06.8 – Other intracranial injuries
	S06.1 – Traumatic cerebral edema
	S06.3 – Focal brain injury
	S06.9 – Intracranial injury unspecified
	S09.7 – Multiple injuries of head
	S09.8 – Other unspecified injuries of head
	S09.9 – Unspecified injury of head
3. Other causes including late effects of injuries poisonings toxic effects & other external causes	T90.2 – Sequelae of fracture of skull and facial bones
	T90.5 – Sequelae of intracranial injury
	T90.8 - Sequelae of other specified injuries of head
	T90.9 – Sequelae of unspecified injury of head
	T96 – Sequelae of poisoning by drugs medicaments and biological substances
	T97 – Sequelae of toxic effects of substances chiefly nonmedicinal as to source
	T98.2 – Sequelae of certain early complications of trauma
**II. Non Traumatic Causes**	
**Diagnosis**	**ICD-10 Code and Description**
1. Toxic effects of substances chiefly non-medical as to source	T51– Toxic effect of alcohol
	T56 – Toxic effect of metals
	T58– Toxic effect of carbon monoxide
2. Complications of surgical & medical care not elsewhere classified	G93.8 Other specified disorders of the brain (including post-radiation encephalopathy)
3. Anoxia	G93.1– Anoxic brain damage (includes all causes of anoxia except those occurring following abortions ectopic pregnancy labour & delivery & newborn)
	T75.1– Drowning and nonfatal submersion
	T71– Asphyxiation suffocation (by strangulation)
4. Vascular insults	I62.0 – Subdural hemorrhage
(Aneurysm and vascular malformations)	I62.9 – Unspecified intracranial hemorrhage
RVL Needs clarification	
Aneurysm I77.0	
Malformation Q28.0 –Q28.9	
Traumatic aneurysm T14.5	
5. Brain Tumours	C71 – Malignant neoplasm of brain
	C79.3 – Secondary malignant neoplasm of brain and cerebral meninges
	D33.0-D33.3 – Benign neoplasm of brain and other parts of central nervous system
	D32.0 – Benign neoplasm of cerebral meninges
	D43 – Neoplasm of uncertain or unknown behaviour of brain and central nervous system
	C70 – Malignant neoplasm of brain
	D43.2 – Neoplasm of brain unspecified
	D42.0 - Neoplasm of uncertain or unknown behaviour of cerebral meninges
6. Encephalitis	A81.1– Subacute sclerosing encephalitis
	B00.4 – Herpesviral meningoencephalitis
	B05.0 – Postmeasles encephalitis
	A83.0 – Japanese encephalitis
	A83.2 – Eastern equine encephalitis
	A86.0 – Unspecified viral encephalitis
	G04.0 – Acute Disseminated encephalitis
	G04.8 – Other encephalitis myelitis and encephalomyelitis
	G04.9 – Encephalitis myelitis and encephalomyelitis unspecified
	B01.1 – Varicella encephalitis
	B02.0 – Zoster encephalitis
	G05. – Encephalitis myelitis and encephalomyelitis in diseases classified elsewhere
7. Metabolic Encephalopathies	E10.0 (Type I) E11.0 (Type II)
	E13.0 – Other specified diabetes mellitus with coma
	E14.0 – Unspecified diabetes mellitus with coma
	E15 – Nondiabetic hypoglycaemic coma
	F07.2 – post concussion syndrome
8. Meningitis	G06.0 – intracranial abscess and granuloma
	G06.1 – Intraspinal abscess and granuloma G06.2 – Extradural and subdural abscess unspecified
	G93.0 – Cerebral cysts
	A87 – Viral meningitis
	B01.0 – Varicella meningitis
	B37.5 – Candidal meningitis
	G00 – Bacterial meningitis not elsewhere classified
	G01* – Meningitis in bacterial diseases classified elsewhere
	G02* – Meningitis in other infectious and parasitic diseases classified elsewhere
	G03. – Meningitis due to other and unspecified causes

### Overview of cost calculation

The focus for this study was the economic burden of patients with ABI in a publicly funded healthcare system. Both total and annualized per patient costs were calculated and reported. Annualized costs were estimated for all ABI patients across a continuum of institutional and community care settings using a bottom-up costing approach. In particular treatment costs incurred in the follow-up period (up to three years after acute care admission) were calculated for acute care emergency department (ED) inpatient rehabilitation (IR) complex continuing care (CCC) home care services and physician visits. Since Ontario has universal public health care insurance program with supplementary workplace drug and disability insurance and private auto insurance all costs in this study were calculated from the government payer’s perspective (Ontario Ministry of Health and Long-Term Care) and expressed in 2007 Canadian dollars. Cost calculation excluded any indirect and direct costs incurred by patient and family such as forgone income or associated out-of-pocket expenses or insurance compensation paid out by third-party payers.

Patient-specific health care utilization data were abstracted from provincial administrative databases and then linked across databases using patients’ scrambled health card identifier. Data on acute hospital admission are stored in the Discharge Abstract Database (DAD) and the National Rehabilitation Reporting System (NRS) contains inpatient rehabilitation data. Data on utilization of homecare services and complex continuing care were obtained from the Home Care Reporting System (HCRS) and the Continuing Care Reporting System (CCRS) respectively. Although the quality of ABI diagnostic coding is insufficient to accurately identify ABI patients in the emergency departments all patients in the cohort were assumed to be admitted to ED prior to their hospitalization. Usage of physician services was identified from Ontario Health Insurance Plan (OHIP).

Unit costs of inpatient acute care ED visits home care and complex continuing care were obtained from the Ministry of Health and Long-Term Care (MOHLTC) Health Data Branch website [28] and are based on costing data submitted by service providers to the MOHLTC. The Health Data Branch provides financial advice to the MOHLTC ensures accountability of health care providers and develops funding allocations to support Ontario health system plans. Complex continuing care costs were derived from government-stipulated payment rates. Ambulatory costs were based on government-stipulated OHIP fee codes for specific service categories using the median reimbursement amount reported in 2007. Rehabilitation costs were based on unit case costs reported in the Joint Planning and Policy Committee’s (JPPC) technical report [29].

Case cost calculation for acute care IR and CCC was based on a case-mix costing methodology in which each patient was assigned a weight representing the intensity of resource utilization during the stay. These patient-specific weights multiplied by a provincial unit cost for a given care setting provide an estimate of the case cost. In acute care and IR settings the unit cost reported the MOHLTC is per weighted case (CPWC) where as in CCC the unit cost is per weighted day. Unit costs for ED home care and physician visits measure the average cost per visit or per hour. Total cost for these services is calculated by multiplying the number of visits or hours of service that the patient utilized by the provincial-average cost.

Total costs were calculated by aggregating individual costs over one, two and three year periods for each type of health care service. Average per patient costs were estimated as unconditional mean by dividing the total costs by the total number of ABI patients in each subgroup at the start of each study period.

#### ED visits and acute care

Emergency department visits were valued at $187 (CAD 2007) which was the average (non-weighted) cost of an ED visit reported by the MOHLTC Health Data Branch [28]. Due to data availability assumptions were required to measure ED utilization by ABI patients. These assumptions were three-fold: 1) all TBI and nTBI patients in the cohort required an ED visit prior to index hospitalization 2) a single ED visit per patient was assumed and additional ED visits throughout a year were not included and 3) mild ABI cases that were discharged home following an ED visit and those that were seen in physician offices only or went undetected were not included in the present study and thus their ED visit costs were unaccounted for. The first assumption is likely to be satisfied for TBI patients but may overestimate ED visits for nTBI patients since some of the nTBI patients could be admitted to hospital for scheduled procedures. Because TBI is of an accidental nature the majority of TBI patients have non-scheduled hospitalizations and thus require an ED visit. Ontario Neurotrauma Foundation (ONF) reported that for TBI patients falls, being struck by and motor vehicle crashes together account for 78.5% of all TBI acute care admissions [30].

The second and third assumptions underestimate total ED costs. The second assumption is not very restrictive since approximately 92% of ABI patients have a single ED episode and only minority of patients have multiple ABI-related ED visits [30]. Also based on our cohort definition the present study omits patients with mild ABI who were treated in the ED but were not admitted to acute care. Among TBI patients there may be many mild cases. Data by ONF suggest that in 2006 there were approximately fifteen thousand TBI patients admitted to ED but only five thousand were treated in acute care [30]. Given the above two assumptions our estimates of ED costs are conservative and provide a lower boundary for ABI-related ED hospitalization costs. Case cost calculation for patients in acute hospitals was based on case-mix methodology described earlier. Hospital patients are categorized into homogeneous case mix groups (CMG) that takes account of patients’ clinical condition or medical procedures. Patient CMG age co-morbidities specific interventions and the length of hospital stay determine his/her resource intensity weight (RIW) that approximates the amount of hospital resources used up during the hospitalization relative to the average inpatient (RIW = 1.0). Following this each case cost is estimated by multiplying the weight by provincial cost per weighted case for acute care valued at $5212 in 2007 [28].

#### Complex continuing care

Each continuing care resident is classified into one of 44 Resource Utilization Groups (RUG-III) according to clinical condition physical functioning and treatment in the last 14 days since admission. Each RUG-III group has an associated Case Mix Index (CMI) (functionally similar to acute care RIWs) that approximates the daily amount of medical resources used to care for residents in that group relative to the average resident. To calculate total CCC case costs we multiplied each patient’s length of stay by the corresponding CMI to measure weighted patient days and then multiplied by the provincial average cost per weighted patient day ($469.2 in 2007).

#### Inpatient rehabilitation

The approach used to calculate rehabilitation-related case costs was similar to the case mix methodology used to calculate acute and CCC costs. Each patient entering the IR facility is assigned to one of the Rehabilitation Patient Groups (RPG) based on the motor and cognitive Functional Independence Measure (FIM) scores age and rehabilitation client code. Patients in each of the RPGs are then given Rehabilitation Cost Weights (RCW) which approximate their resource intensity relative to the average rehabilitation patient. Individual rehabilitation case cost is calculated by multiplying each patient’s weight by the rehabilitation cost per weighted case (CPWC). Unlike acute CPWC rehabilitation CPWC is not reported every year. The rehabilitation CPWC used in this study was based on costs reported by JPPC for the 2004/05 fiscal year and then extrapolated to 2007 using the relative changes in acute hospital case costs [29].

#### Home care

Total costs for home care services were estimated using patient’s total annual number of visits or hours in each of 14 service categories multiplied by the average provincial costs for each service reported by the MOHLTC. Per patient average annual cost was estimated by dividing total costs by the number of patients in each subgroup.

#### Ambulatory care

Although this has recently begun to change traditionally primary care physicians in Canada have been paid according to a fee-for-service model [31,32]. Fee-for-service physician costs were obtained based on the total number of visits by service type and the median reimbursed amount for the associated fee codes in 2007 fiscal year. Annualized per patient costs were calculated by dividing the cumulative cost by the total number of patients in each subgroup.

### Privacy and ethics

This study received ethics approval from the Toronto Rehabilitation Institute Research Ethics Board. All investigators and staff involved in the study signed confidentiality agreements and analyses were conducted with de-identified data.

## Results

The characteristics of the TBI and nTBI cohorts can be found in Tables 2 and 3. Overall 10% of the TBI cohort and 9% of the nTBI cohort were discharged to rehabilitation. The sex distribution was similar for both discharge destinations in each of the cohorts. In both cohorts older patients were more likely to be discharged to rehabilitation compared to younger patients. In addition those discharged to rehab were more likely to live in non-rural areas and have longer inpatient lengths of stay. Among the TBI cohort those discharged to rehabilitation were more likely to have higher Charlson Comorbidity Index scores and to have been caused by a motor vehicle collision. Among the nTBI cohort the majority of patients had a brain tumor as a diagnosis (60%) among those discharged to rehabilitation; there was a higher percentage that had brain tumour (67%) and vascular insults (13%) as a diagnosis. The identified diagnostic categories for TBI patients were broader and more heterogeneous. Just over 32% of patients were represented in more than one diagnosis category. nTBI patient diagnostic categories were more distinct with only 0.9% represented in more than one diagnosis category.

**Table 2:** Demographic and characteristics of TBI Cohort

	**Discharged to Rehab [N (%)]**	**Not discharged to Rehab [N (%)]**	**Total [N (%)]**
Sex			
Female	403 (34)	3717 (34)	4120 (34)
Male	784 (66)	7066 (66)	7850 (66)
Age			
<18	21 (2)	2460 (23)	2481 (21)
18-34	262 (22)	1891 (18)	2153 (18)
35-54	306 (26)	2114 (20)	2420 (20)
55-64	144 (12)	1040 (10)	1184 (10)
65-74	133 (11)	1061 (10)	1194 (10)
75+	321 (27)	2217 (21)	2538 (21)
Residence			
Non-Rural	1016 (86)	8570 (80)	9586 (80)
Rural	171 (14)	2213 (20)	2384 (20)
Charlson Comorbidity Index			
0-1	1035 (87)	10018 (93)	11053 (92)
2-3	132 (11)	615 (6)	747 (6)
4+	20 (2)	150 (1)	170 (1)
Inpatient LOS (days)			
1-2	5 (0)	2760 (25)	2765 (23)
3-5	56 (5)	2099 (29)	3155 (26)
6-11	224 (19)	2451 (23)	2675 (22)
12+	902 (76)	2473 (23)	3375 (28)
Motor Vehicle Related			
Yes	416 (35)	2099 (20)	2515 (21)
No	771 (65)	8684 (80)	9455 (79)
TBI category*			
Fracture of Skull	440 (37)	3257 (30)	3697 (30)
Intercranial Injury	1098 (92)	8916 (82)	10014 (83)
Other causes	30 (2)	670 (6)	700 (5)

* Categories are not mutually exclusive 32% of patients have more than one listed diagnosis.

**Table 3:** Demographic and characteristics of nTBI Cohort

	**Discharged to Rehab [N (%)]**	**Not discharged to Rehab [N (%)]**	**Total [N (%)]**
Sex			
Female	1308 (47)	13741 (48)	15049 (48)
Male	1496 (53)	14956 (52)	16452 (52)
Age			
<18	10 (0)	2450 (9)	2460 (8)
18-34	106 (4)	2235 (8)	2341 (7)
35-54	485 (17)	6056 (21)	6541 (21)
55-64	450 (16)	4519 (16)	4969 (16)
65-74	644 (23)	5298 (18)	5942 (19)
75+	1109 (40)	8129 (28)	9248 (29)
Residence			
Non-Rural	2444 (87)	23856 (83)	26300 (84)
Rural	360 (13)	4841 (17)	5201 (16)
Charlson Comorbidity Index			
0-1	1445 (52)	14675 (51)	16120 (51)
2-3	904 (32)	6885 (24)	7789 (25)
4+	455 (16)	7137 (25)	7582 (24)
Inpatient LOS (days)			
1-2	24 (1)	2911 (10)	2935 (9)
3-5	168 (6)	6943 (24)	7111 (23)
6-11	584 (21)	8148 (28)	8732 (28)
12+	2028 (72)	10695 (37)	12723 (40)
Type of nTBI*			
Brain Infection	274 (10)	4023 (14)	4297 (14)
Brain Tumour	1888 (67)	17172 (60)	19060 (60)
Metabolic encephalopathies	109 (3)	2086 (7)	2195 (7)
Anoxia	121 (4)	902 (3)	1023 (3)
Complications of Surgical and Medical Care	74 (3)	754 (3)	828 (3)
Vascular Insults	358 (13)	1065 (6)	2423 (8)
Toxic Effects	12 (0)	1918 (7)	1930 (6)

* Categories are not mutually exclusive 0.9% of patients have more than one diagnosis.

A detailed breakdown of the average direct per-patient costs appears in Table 4. Several important features of the cost structure emerge from the table. Treatment of nTBI patients and of patients discharged to rehabilitation facility is more expensive relative to TBI patients and patients not discharged for rehabilitation respectively in the first year and in subsequent years. Although costs decrease with time since the injury they remain substantial three years after the index hospitalization. The estimated average direct medical cost in the first year following an acute care admission for TBI and nTBI patients was $32132 and $38018 respectively. ABI patients who required rehabilitation had considerably higher costs than those who were discharged from acute to other destinations; a result that holds for both traumatic ($93340 versus $25394) and non-traumatic injuries ($82241 versus $33697). This was not only due to the fact that these patients utilized more rehabilitation services but also because they had higher overall treatment costs for physician home care and other health care services.

**Table 4:** Mean direct costs per ABI patient across health care settings in the first 3 years since injury

	**TBI**			**nTBI**		
	**All TBI Pts.**	**DC to IR**	**Not DC to IR**	**All nTBI Pts.**	**DC to IR**	**Not DC to IR**
**Year 1 [N]**	[11970]	[1187]	[10783]	[31501]	[2804]	[28697]
	Mean Annual Cost (standard deviation)			Mean Annual Cost (standard deviation)		
Emergency Department	**$187**	**$187**	**$187**	**$187**	**$187**	**$187**
Acute Care	**$19083**	**$43123**	**$16436**	**$23078**	**$37953**	**$21625**
	(72495)	(43441)	(74538)	(77157)	(45451)	(79431)
Inpatient Rehabilitation	**$5363**	**$33646**	**$2250**	**$3149**	**$23610**	**$1150**
	(22191)	(43062)	(15649)	(13379)	(29211)	(8261)
Complex Continuing Care	**$3535**	**$8168**	**$3025**	**$5626**	**$11855**	**$5017**
	(28259)	(40179)	(26576)	(32105)	(49895)	(29734)
Homecare	**$722**	**$993**	**$692**	**$1626**	**$2104**	**$1580**
	(2259)	(2439)	(2236)	(3856)	(3793)	(3859)
Physician	**$3242**	**$7223**	**$2804**	**$4352**	**$6532**	**$4138**
	(5122)	(8152)	(4458)	(4251)	(4702)	(4143)
**Annual Cost**	**$32132**	**$93340**	**$25394**	**$38018**	**$82241**	**$33697**
**Year 2 [N]**	[11294]	[1107]	[10187]	[23049]	[2372]	[20677]
Emergency Department	**$3**	**$5**	**$3**	**$9**	**$8**	**$9**
Acute Care	**$377**	**$462**	**$368**	**$787**	**$689**	**$798**
	(5434)	(3894)	(5573)	(6027)	(5855)	(6047)
Inpatient Rehabilitation	**$148**	**$464**	**$114**	**$127**	**$381**	**$98**
	(3720)	(5776)	(3428)	(2545)	(4664)	(2177)
Complex Continuing Care	**$606**	**$1219**	**$540**	**$1018**	**$2067**	**$898**
	(11297)	(18599)	(10192)	(13093)	(19010)	(12228)
Homecare	**$512**	**$892**	**$471**	**$1242**	**$1934**	**$1162**
	(2436)	(3705)	(2251)	(4146)	(5068)	(4019)
Physician	**$934**	**$1325**	**$892**	**$1515**	**$1790**	**$1483**
	(1607)	(1600)	(1602)	(2226)	(3076)	(2104)
**Annual Cost**	**$2580**	**$4367**	**$2388**	**$4698**	**$6869**	**$4448**
**Year 3 [N]**	[9068]	[897]	[8171]	[17325]	[1799]	[15526]
Emergency Department	**$1**	**$1**	**$1**	**$4**	**$4**	**$4**
Acute Care	**$116**	**$287**	**$98**	**$263**	**$350**	**$254**
	(2542)	(4935)	(2154)	(2915)	(3482)	(2845)
Inpatient Rehabilitation	**$0**	**$0**	**$0**	**$83**	**$169**	**$74**
				(2056)	(2694)	(1971)
Complex Continuing Care	**$483**	**$344**	**$498**	**$1045**	**$2120**	**$922**
	(6698)	(3675)	(6944)	(12303)	(15052)	(11954)
Homecare	**$606**	**$1086**	**$555**	**$1378**	**$2305**	**$1271**
	(3695)	(4633)	(3573)	(5596)	(6520)	(5469)
Physician	**$1028**	**$1433**	**$984**	**$1625**	**$1901**	**$1593**
	(2315)	(5188)	(1744)	(2720)	(2751)	(2715)
**Annual Cost**	**$2234**	**$3151**	**$2136**	**$4398**	**$4544**	**$2847**

**Figure 1:**
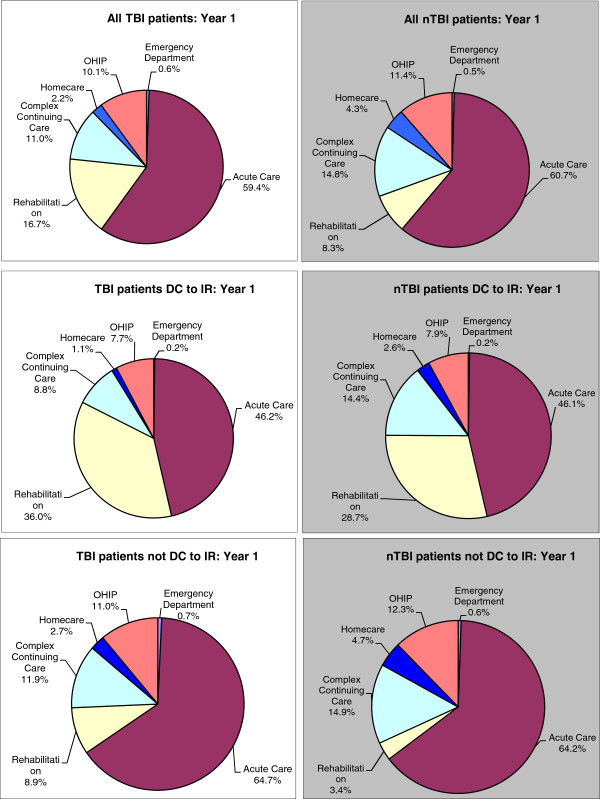
Cost distribution across health care system in the first year since discharge (DC).

In the first year post-injury the initial hospital stay accounted for the largest cost in all sub-groups of patients. For TBI patients discharged to IR cost associated with acute care stay made up 46% of the total per-patient cost followed by rehabilitation (36%) and CCC (9%). Acute care cost for TBI patients not discharged to inpatient rehabilitation although smaller in magnitude was the largest contributor to total cost (65%). CCC accounted for 12% of the total per-patient cost among those not discharged to inpatient rehabilitation. The distribution of costs was very similar for TBI and nTBI patients once the discharge destination was taken into account (Figure 1).

The mean annualized treatment costs decreased dramatically in second year post injury with expenditure per TBI and nTBI patient being $2580 and $4698 respectively. Ongoing homecare physician and CCC care accounted for proportionally more costs in the second and third year. Ninety-one percent of TBI patients and 88% of nTBI patients (data not shown) utilized physician services in the second year which accounted for 36% and 68% of the respective total mean cost. Only 4.5% and 1.8% of TBI and nTBI patients were readmitted to acute care in the second year. There was a very slight decrease in the total average costs in the third year relative to the second for both TBI and nTBI patients with per patient cost remaining above two- and four thousand dollars respectively.

## Discussion

Canada is among other developed countries that are currently facing the challenge of controlling their publicly funded health care expenses and mounting fiscal debt. In 2008 health care expenditure totalled CAD $141.4 billion making up 10.7% of Canadian GDP and it is projected to continue to increase in the future [33]. In light of these challenges policy- and decision-makers have turned their attention to cost-of-illness (COI) studies to help direct attention to areas for improving health care accountability and long-term sustainability of health care expenditures [34,35]. The present study highlights considerable individual and total system costs for the treatment of ABI patients in Canada.

This population-based study followed a cohort of ABI patients discharged from acute care in 2004 – 2008 and examined the direct cost of publicly financed health care services in the first 3 years since the brain injury. It presented comprehensive information on direct costs of ABI by including costs across the entire health care system such as ED and acute inpatient costs, the costs of rehabilitation following the index event as well as CCC home care and physician costs. It also uses recent Canadian data and is the first costing study of ABI conducted on such wide scale and scope. Our main findings are four-fold: 1) treatment of nTBI patients is more expensive than of TBI patients both per patient and on aggregate; 2) discharge to inpatient rehabilitation is associated with higher treatment costs; 3) health care costs decrease over time but remain significant even after 3 years; and 4) for all subgroups of patients the highest cost in the first year was acute care while the highest three costs in the second and third year were physician and home care services and CCC.

Mean cost of treatment for an nTBI patient was only 18% higher than that for a TBI patient in the first post-injury year; however in the second and third follow-up years nTBI costs were nearly double those for TBI (Table 2). This suggests that on average nTBI patients suffer longer lasting health impairments and require substantially more intensive ongoing care than patients with TBI. The costs in the first year post-injury for TBI patients are mainly driven by intensive utilization of acute care rehabilitation and CCC services which corroborates with findings reported by Canadian Institute of Health Information (CIHI) [10,11]. CIHI estimated that that the median LOS in rehabilitation and CCC facilities for patients with head injury was more than two times that of an average patient. Earlier analyses of a similar population also showed that intensity of acute care (length of stay special care days) were positively associated with inpatient rehabilitation. [36] For patients with nTBI first-year costs were largely influenced by the cost of acute care CCC and physician visits.

Patients discharged to inpatient rehabilitation were more costly that those who were not. These findings are consistent with other studies that linked injury severity to higher treatment costs [11 20 23 36]. Brooks et al. [20] grouped patients by severity in two ways: based on the Abbreviated Injury Scale (AIS) and by patient’s rehabilitation discharge status. Brooks and colleagues found that costs increased with injury severity for both severity metrics. Vangel et al. [11] found that motor deficiency was a significant predictor of higher future medical billings. The study by Brooks et al. reported that the first year cost including initial hospitalization and follow-up services was $151149 ($228626 in 2007 dollars) per TBI patient discharged to IR and $16813 ($25431 in 2007 dollars) per TBI patient not discharged to IR. Our cost estimate for TBI patients not discharged to IR is slightly above the one reported by Brooks et al. potentially in part because we capture costs for patients admitted to IR at a later point in the year while Brooks et al. did not allow for this. On the other hand our cost per TBI patient discharged to IR is significantly below Brooks’. The latter disparity may be due to the fact that the study by Brooks et al. was based on US data and potentially reflects the difference in health care costs and provision between Canada and the US; in particular the use of technologies and rehabilitation services may be relatively more intensive and expensive in the US. Overall the cost of treatment of patients with ABI in the first year post-injury is significant and comparable in magnitude to other health care conditions such as cardiac arrhythmia ($22000), stroke ($34000) and hip fracture ($35000) [37]. A report to the Ontario Neurotrauma Foundation estimated approximately five thousand TBI and eleven thousand nTBI related hospitalizations in Ontario per year [30]. Total medical costs for TBI and nTBI patients in Ontario in the first follow-up year are approximately $120.7 and $368.7 million respectively with acute care cost overwhelming all other cost components. Assuming that incidence of brain injury in Canada is similar to that of Ontario implies that direct total annual costs in Canada for TBI and nTBI treatment in the first year amount to $331.1 and $1077.4 million respectively. Citing the Public Health Agency of Canada (PHAC) CIHI reported that the direct cost of head injury in Canada in 2000–2001 was CAD $151.7 million [16]. This represents approximately 46% of our estimate of the total cost of TBI in Canada however in their calculation PHAC did not include the cost of rehabilitation and complex continuing care which as we have shown can be substantial.

The present study while rigorous in its methodological approach has certain limitations. Cost calculation was restricted to major health care service categories paid for by the Ontario government. It excluded some government payments for pre-hospital costs diagnostic services, outpatient drugs and supporting equipment and supplies. In this study we took government payer perspective and therefore excluded cost of services paid through private insurance or out-of-pocket. Only direct medical costs were estimated and indirect costs in terms of forgone income of patient or caregiver reduced productivity and disability were unaccounted for. Given that brain injury often leads to lingering health consequences impairments and disability, indirect costs are likely to outweigh the direct ones. Grabow et al. [38] previously assessed indirect costs of head injury through patients’ interviews and determined that they accounted for 92% of the total annual costs while Max and colleagues [39] reported that wages lost due to disability or death represented 88% of the total cost. Therefore direct medical costs represent only a small fraction of the total burden of ABI. Finally this study included all health system costs and not only those attributable exclusively to ABI. The latter would require eliminating the costs incurred due to health condition other than the primary condition of interest and requires a control group that was not available to us for this study. We also limited our study population to ABI patients who were initially admitted to an acute care hospital and do not include costs of treating patients with mild ABI who are not admitted to hospital since ABI diagnostic coding in the emergency department and outpatient setting is insufficient to accurately include these patients. We also included all patients with any diagnosis that would include all causes of injury including patients who suffered an ABI by any hospital process such as elective surgery. Though we do not have information on the present cohort prior analysis of a similar cohort [36] indicated that 1.8% of TBI patients and 3.2% of nTBI patients were not primarily ABI-admissions. This is a small margin of inclusiveness in defining the present cohort. All individuals in the cohort require treatment for ABI in the health care system. Finally we did not have functional status and were unable to assess the direct costs associated with greater functional impairment directly and rely instead on admission to inpatient rehabilitation to proxy for greater levels of impairment.

Despite the above-mentioned limitations the current study has several important strengths. It provided recent and comprehensive estimate of direct medical costs of ABI across a wide continuum of institutional and community health care settings. This is the first study on direct cost of health care services utilized by patients with nTBI and is only a second study of costs associated with TBI in Canada. Cost estimated in the previous study by Snow et al. [40] were based on patients admitted to a single hospital Sunnybrook Medical Center based in Toronto Ontario and only included hospitalization costs. The use of Ontario administrative healthcare databases permitted us to obtain population-based cost estimates across the entire health care system that are more precise and detailed thus addressing the methodological weaknesses characteristic of other studies [10,11,20,40,41]. Finally the longitudinal nature of the study allowed us to examine the evolution of patient costs and utilization patterns over time which could be used for appropriate planning of health care expenses for ABI patients. Future studies however should include cost estimates that capture persons with ABI who are seen in the emergency room or by physician only as well as the indirect costs. In addition cost analysis could be expanded to statistically analyse what factors contribute most to the economic burden of nTBI and TBI including comorbidities or markers of severity and in comparison to a non-ABI control-group matched on health and functional status. This would be enabled by more fulsome individual data from charts or prospective data collection.

## Conclusions

The main finding of this study is that direct medical costs in the ABI population are substantial with mean cost in the first year post-injury per TBI and nTBI patient being $32132 and $38018 respectively. Although most expenses occur in the first follow-up year ABI patients continue to use medical services in the second and third year with emphasis shifting from acute care and rehabilitation towards homecare physician services and CCC. Understanding the clinical and health system factors influencing health care utilization in this population and a degree to which health care costs can be controlled is important for planning prevention programs and reducing cost of care among these patients. The data presented here are useful for policy planning and cost-effectiveness analysis.

## Competing interests

The authors declare that they have no competing interests.

## Authors’ contributions

AChen and AColantonio conceived and designed the study and acquired the data. DP provided comments and helped to conceive the study. KB and AChen prepared the initial draft of the manuscript. WW and KB interpreted the results and developed the costing methodology. BZ performed the statistical data analysis. All authors critically reviewed read and approved the final manuscript before submission.

## Pre-publication history

The pre-publication history for this paper can be accessed here:

http://www.biomedcentral.com/1471-2377/12/76/prepub
